# Crystal structure and Hirshfeld surface analysis of *N*,*N*′-(2,2-di­chloro-3-oxo-3-phenyl­propane-1,1-di­yl)diacetamide

**DOI:** 10.1107/S205698902500670X

**Published:** 2025-08-05

**Authors:** Firudin I. Guseinov, Fatali E. Huseynov, Ksenia A. Afanaseva, Bogdan I. Ugrak, Lev M. Glukhov, Aida I. Samigullina, Mehmet Akkurt, Gizachew Mulugeta Manahelohe

**Affiliations:** a Kosygin State University of Russia, 117997 Moscow, Russian Federation; b N. D. Zelinsky Institute of Organic Chemistry, Russian Academy of Sciences, 119991 Moscow, Russian Federation; c Department of Ecology and Soil Sciences, Baku State University, Z. Xalilov Str. 23, Az 1148 Baku, Azerbaijan; d Department of Physics, Faculty of Sciences, Erciyes University, 38039 Kayseri, Türkiye; e Department of Chemistry, University of Gondar, PO Box 196, Gondar, Ethiopia; Harvard University, USA

**Keywords:** crystal structure, α,α-dihalogen-β-oxoaldehydes, hydrogen bonds, C—H⋯π inter­actions, Hirshfeld surface analysis

## Abstract

The conformation of the title mol­ecule, C_13_H_14_Cl_2_N_2_O_3_, is maintained by intra­molecular N—H⋯O, C—H⋯O, and C—H⋯Cl inter­actions, creating *S*(6), *S*(5), and *S*(6) motifs, respectively. In the crystal, inter­molecular N—H⋯O, C—H⋯O, and C—H⋯Cl inter­actions connect the mol­ecules, forming a three-dimensional network. Additionally, the mol­ecules are linked by C—H⋯π inter­actions, forming layers parallel to the (002) plane. The most important inter­actions, according to Hirshfeld two-dimensional fingerprint plots, are H⋯H (35.0%), O⋯H/H⋯O (21.2%), Cl⋯H/H⋯Cl (20.7%), and C⋯H/H⋯C (17.1%).

## Abstract 2

In the crystal, mol­ecules are inter­connected by inter­molecular N—H⋯O, C—H⋯O, and C—H⋯Cl inter­actions establishing a three-dimensional network. Furthermore, the mol­ecules form layers parallel to the (002) plane *via* C—H⋯π inter­actions.

## 1. Chemical context

**Figure 1:**
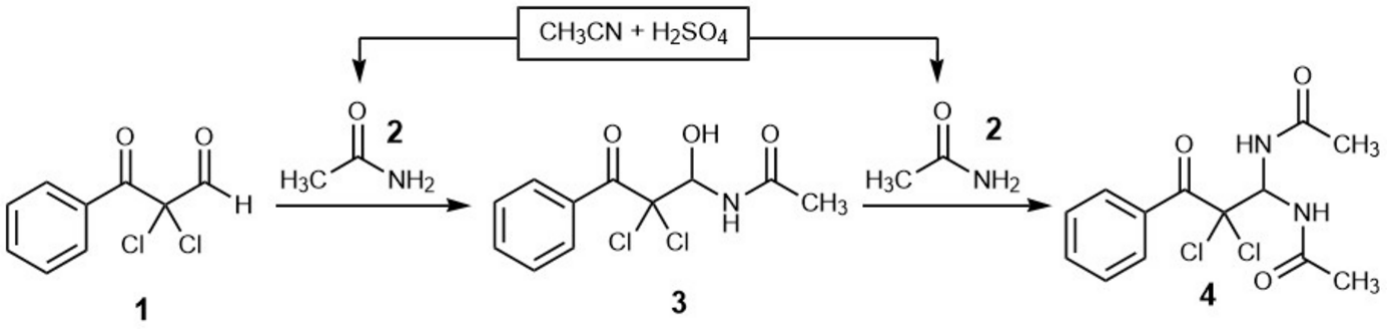
Synthesis of *N*,*N*′-(2,2-di­chloro-3-oxo-3-phenyl­propane-1,1-di­yl)diacetamide.

Bisamidals are an important class of organic compounds, since the amide fragment is a component of many biologically active substances and is widely used in pharmaceuticals, medicine and materials science (Manne *et al.*, 2017 ▸; Zhang *et al.*, 2013 ▸). Bisamidals are also convenient starting reagents for the synthesis of heterocyclic and organo­phospho­rus compounds with useful properties (Dmitriev *et al.*, 2021 ▸; Makra *et al.*, 2022 ▸). The catalytic and analytic properties of this class of compounds are strongly dependent on the attached groups to the amide moiety (Alieva *et al.*, 2006 ▸; Aliyeva *et al.*, 2024 ▸). Both the NH and C=O groups of bis­amidals can participate in various sorts of inter­molecular inter­actions, which improve the catalytic and biological activity of corresponding metal complexes (Kopylovich *et al.*, 2012*a* ▸,*b* ▸; Mahmudov *et al.*, 2015 ▸). We have previously shown that accessible highly electrophilic *α*,*α*-dihalogen-*β*-oxo­aldehydes readily condense with amides to form amidals (Guseinov *et al.*, 1994 ▸,2024 ▸ and 2025 ▸). We used this property of aldehydes (**1**) to obtain bis­amidals (**4**). We found that bis­amidals can be synthesized with a yield of 92% by reacting aldehydes with aceto­nitrile in the presence of concentrated sulfuric acid at room temperature. The formation of product (**4**) occurs *via* amide (**2**) and amidals (**3**) according to the scheme shown in Fig. 1 ▸. The structure of the product (**4**) was proven by NMR spectroscopy and X-ray diffraction.
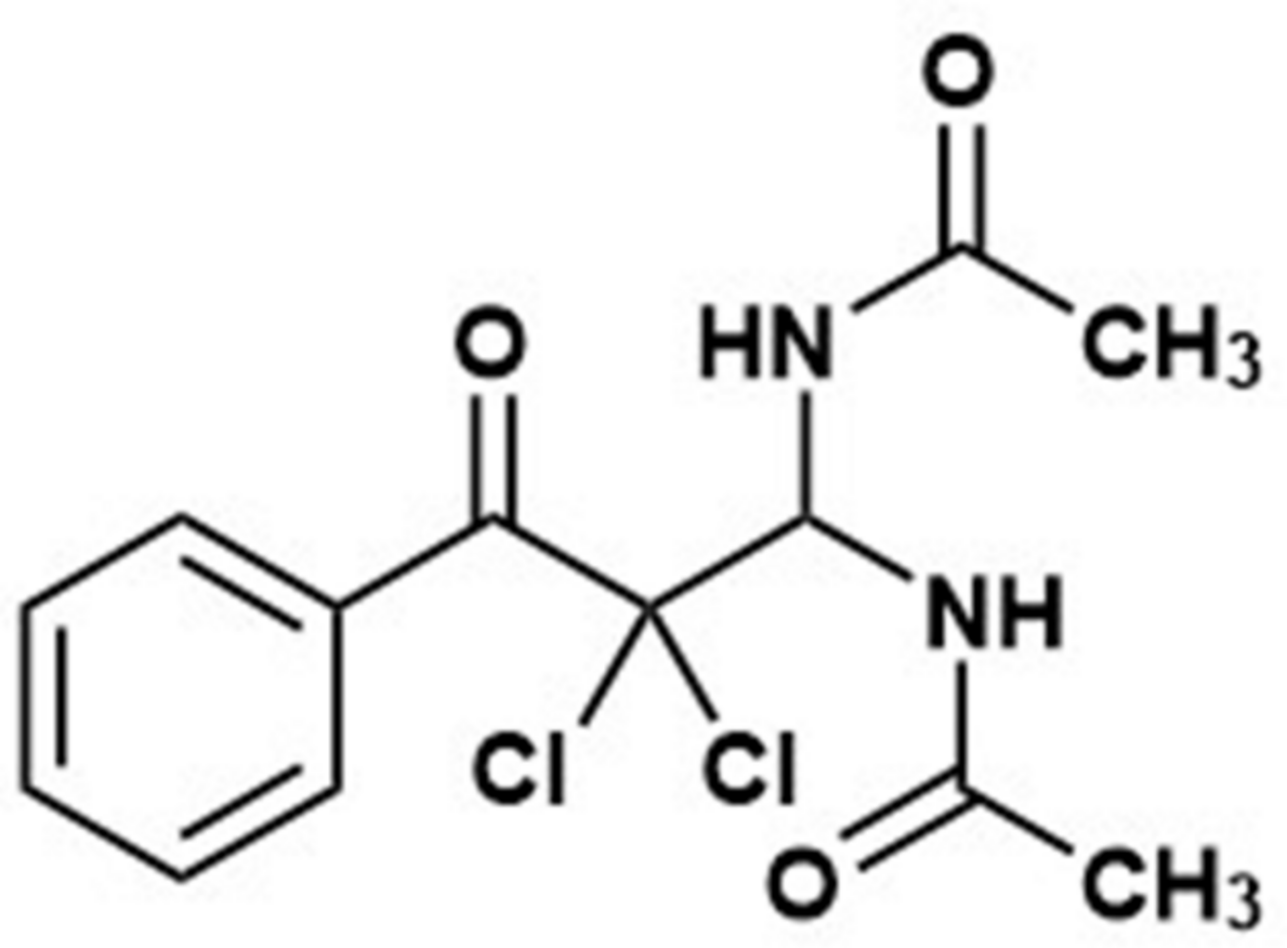


## 2. Structural commentary

**Figure 2:**
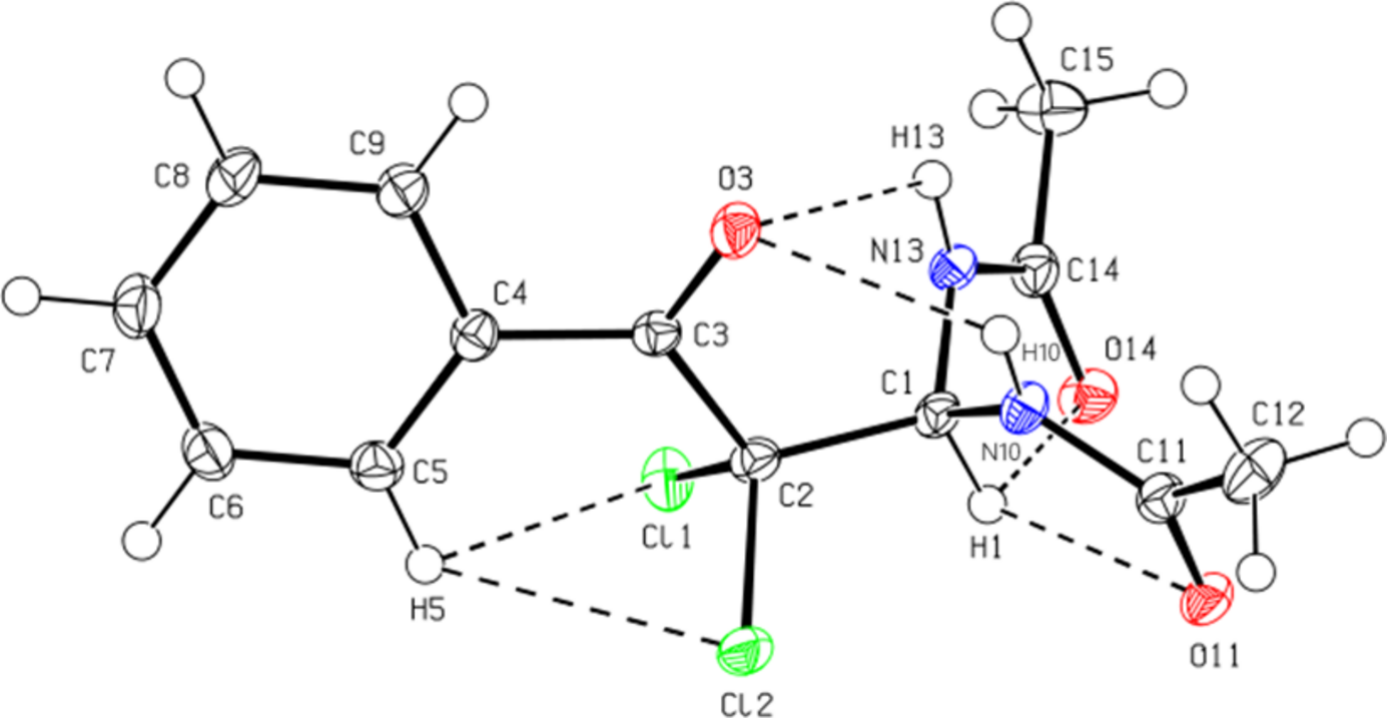
The mol­ecular structure of the title compound with the atom labelling and displacement ellipsoids drawn at the 50% probability level.

As shown in Fig. 2 ▸, the mol­ecular conformation is not planar. Intra­molecular N—H⋯O, C—H⋯O, and C–H⋯Cl inter­actions maintain the mol­ecular conformation, forming *S*(6), *S*(5), and *S*(6) motifs (Bernstein *et al.*, 1995 ▸), respectively. The C9—C4—C3—O3, C9—C4—C3—C2, C4—C3—C2—Cl1, C4—C3—C2—Cl2, C3—C2—C1—N10, C3—C2—C1—N13, C2—C1—N10—C11 and C2—C1—N13—C14 torsion angles are 14.8 (3), −162.76 (19), 46.2 (2), −73.6 (2), 63.4 (2), −64.2 (2), 125.26 (18) and −119.66 (19)°, respectively. The mol­ecule exhibits no unusual bond lengths or inter-bond angles.

## 3. Supra­molecular features and Hirshfeld surface analysis

**Figure 3:**
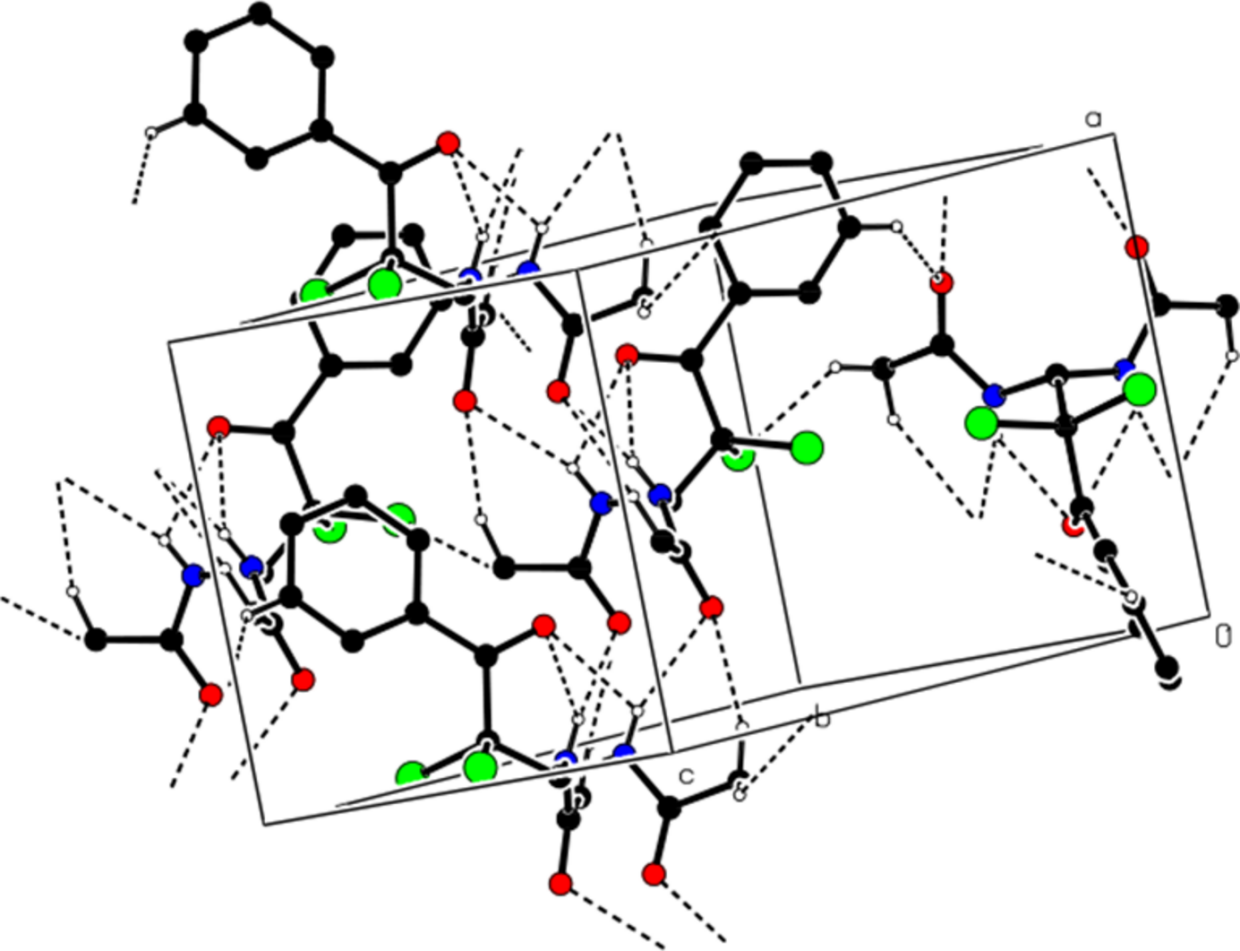
A partial view of mol­ecular packing in the unit cell formed by inter­molecular N—H⋯O, C—H⋯O and C—H⋯Cl hydrogen bonds. Hydrogen atoms that are not involved in these inter­actions have been omitted for clarity.

**Figure 4:**
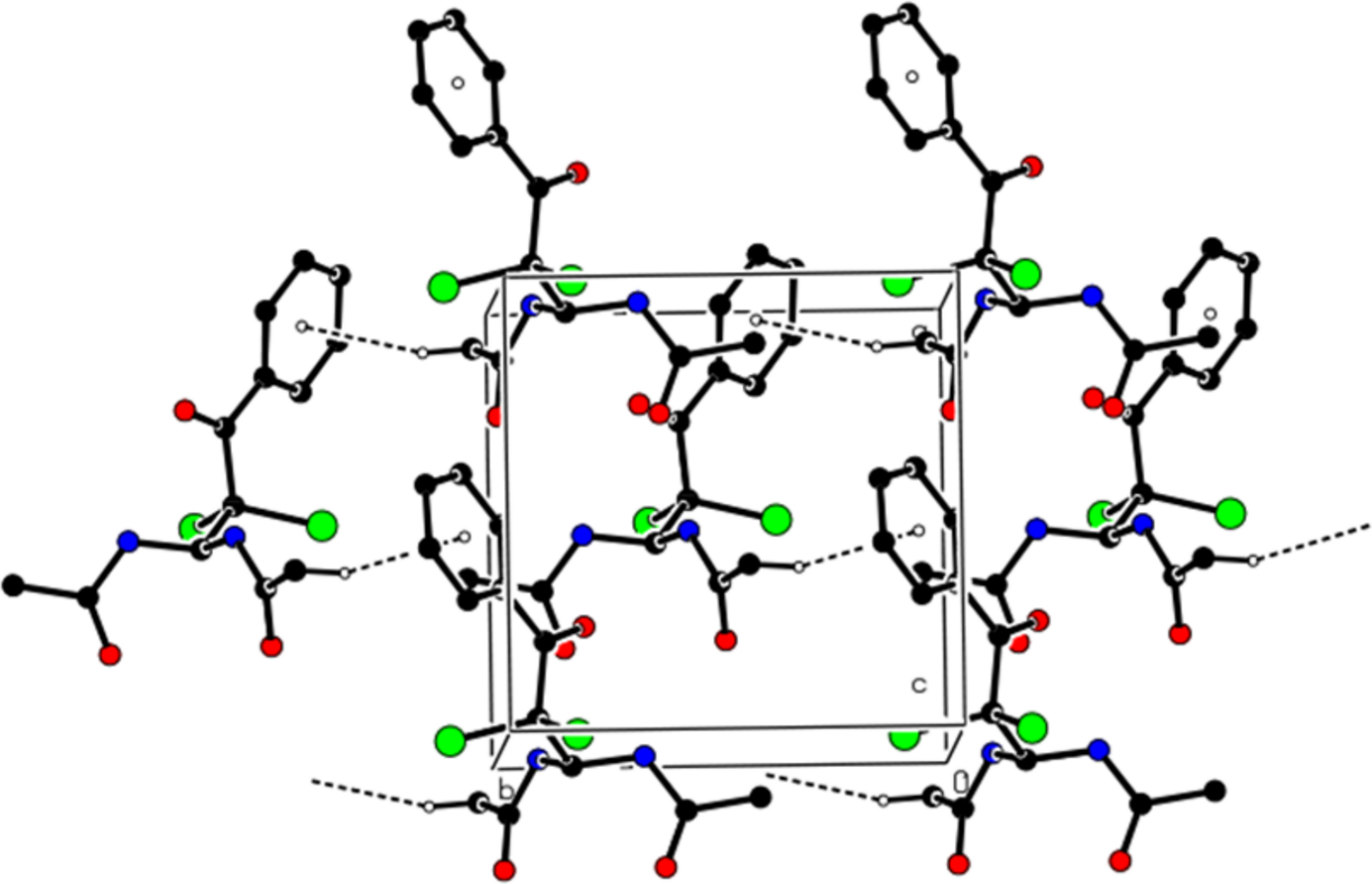
The view of the packing formed by C—H⋯π hydrogen bonds in the unit cell. H atoms that are not involved in these inter­actions have been removed for clarity.

In the crystal, the mol­ecules are linked into a three-dimensional network by inter­molecular N—H⋯O, C—H⋯O, and C—H⋯Cl inter­actions (Table 1 ▸, Fig. 3 ▸). In addition, the mol­ecules create layers parallel to the (002) plane through C—H⋯π inter­actions (Table 1 ▸, Fig. 4 ▸). No π–π inter­actions are observed in the structure.

**Figure 5:**
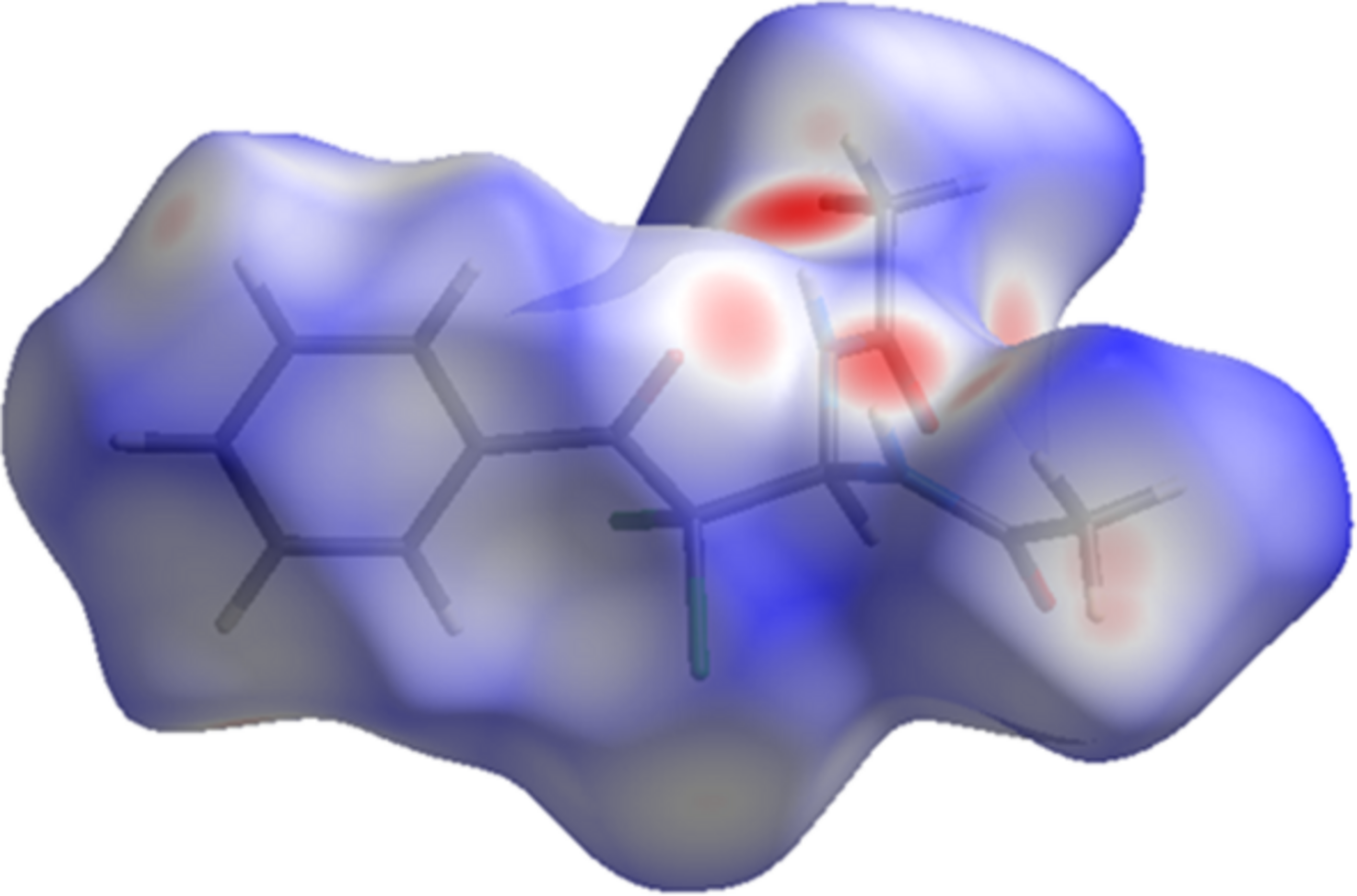
A view of the three-dimensional Hirshfeld surface of the title compound mapped over *d*_norm_.

**Figure 6:**
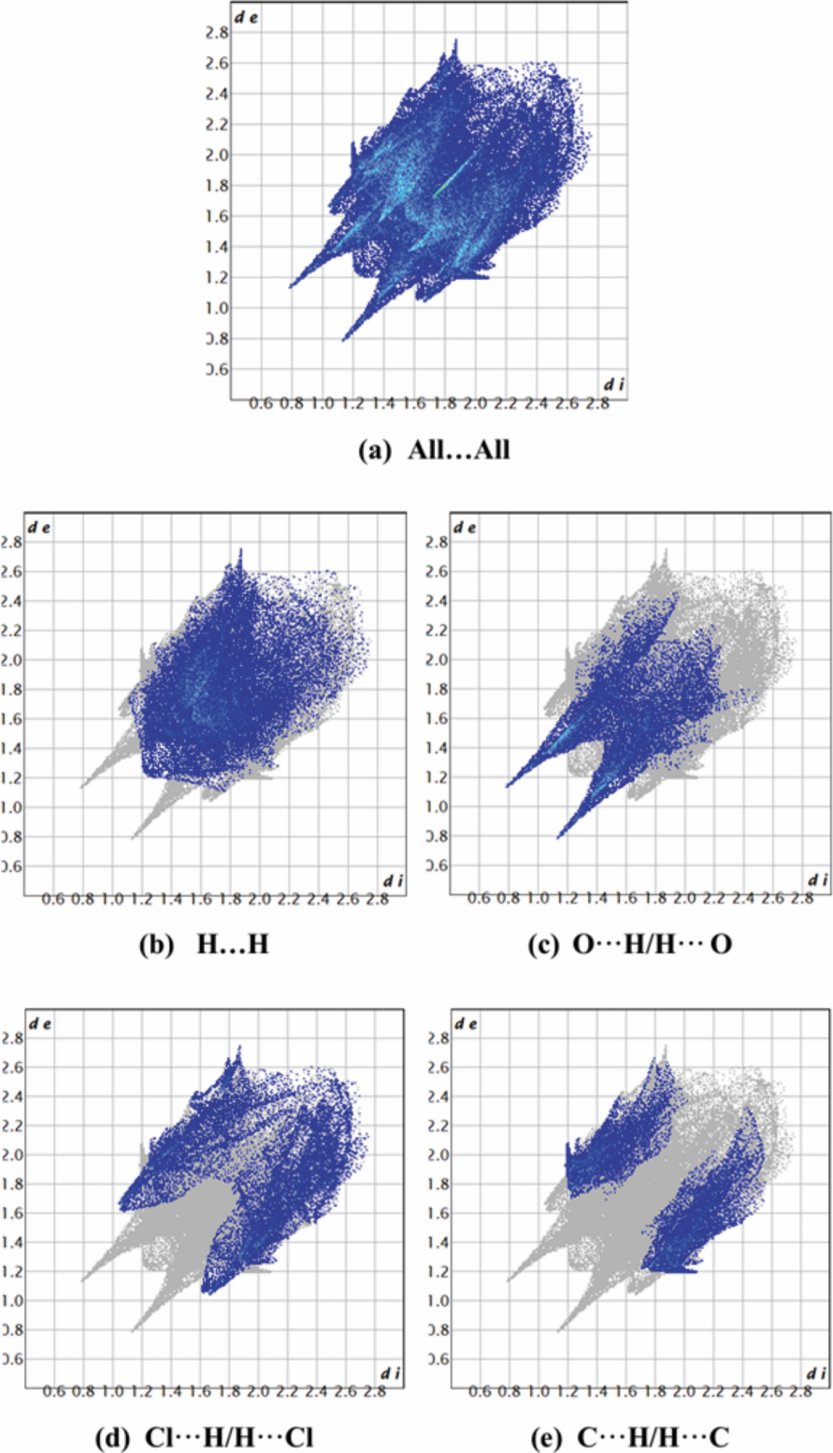
The full two-dimensional fingerprint plots for the title compound, showing (*a*) all inter­actions, and delineated into (*b*) H⋯H, (*c*) O⋯H/H⋯O, (*d*) Cl⋯H/H⋯Cl and (*e*) C⋯H/H⋯C inter­actions. The *d*_i_ and *d*_e_ values are the closest inter­nal and external distances (in Å) from given points on the Hirshfeld surface.

The inter­molecular inter­actions (Tables 1 ▸ and 2 ▸) in the title compound were analysed using Hirshfeld surface calculations, employing *CrystalExplorer 17.5* (Spackman *et al.*, 2021 ▸). The Hirshfeld surface plotted over *d*_norm_ is shown in Fig. 5 ▸. The two-dimensional fingerprint plots (Fig. 6 ▸) show that the most significant contacts are H⋯H (35.0%), O⋯H/H⋯O (21.2%), Cl⋯H/H⋯Cl (20.7%), C⋯H/H⋯C (17.1%), Cl⋯Cl (2.1%), O⋯C/C⋯O (2.0%), O⋯N/N⋯O (0.9%), O⋯O (0.7%) and N⋯H/H⋯N (0.2%).

## 4. Database survey

A search of the Cambridge Structural Database (CSD, Version 6.00, update of April 2025; Groom *et al.*, 2016 ▸) for the 2,2-di­chloro-1-phenyl­propan-1-one unit generated 51 hits, the four most closely related to the title compound being those with refcodes QIRPUG (Clegg & Harrington, 2023 ▸), UHIQUZ (Essa *et al.*, 2015 ▸), UHIROU (Essa *et al.*, 2015 ▸) and YUXMIN (Mamedov *et al.*, 1995 ▸).

QIRPUG and YUXMIN crystallize in the monoclinic space group *P*2_1_/*n*, while UHIQUZ and UHIROU crystallize in the triclinic space group *P*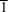.

In the crystal of QIRPUG, the mol­ecules are linked into a three-dimensional network by O—H⋯O and C—H⋯O inter­actions. In addition, π–π and C—H⋯π inter­actions are also observed. In UHIQUZ, the mol­ecules are linked into layers parallel to the (010) plane by N—H⋯O and O—H⋯F inter­actions. The structure also contains π–π and C—H⋯π inter­actions. In UHIROU, the mol­ecules linked by C—H⋯O and O—H⋯Cl inter­actions form layers parallel to the (001) plane. The structure also exhibits π–π and C—H⋯π inter­actions. In YUXMIN, the mol­ecules connect through C—H⋯O inter­actions, forming a three-dimensional network and C—Cl⋯π inter­actions are also observed.

## 5. Synthesis and crystallization

To a solution of 217 mg (1 mmol) of 2,2-di­chloro-3-oxo-3-phenyl­propanal in 10 ml of aceto­nitrile was added sulfonic acid (2 mmol) at room temperature. The reaction mixture was then stirred for 1h. The solvent was removed *in vacuo*, the remaining white powder was recrystallized from chloro­form and *N*,*N′*-(2,2-di­chloro-3-oxo-3-phenyl­propane-1,1-di­yl)di­acetamide was isolated. Yield 292 mg (92%); m.p. 378–380 K. Analysis calculated (%) for C_13_H_14_Cl_2_N_2_O_3_: C 49.23, H 4.45, N 8.83, found C 45.18, H 4.41, N 8.82. ESI–MS: 316.0410. ^1^H NMR (300 MHz, DMSO-*d*_6_): 1.87 (6H, 2CH_3_), 6.95-8.10 (5H, Ar), 8.4 (2H, 2NH). ^13^C NMR (75 MHz, DMSO-*d*_6_): 22.24, 60.28, 89.93, 128.46, 129.83, 131.99, 133.77, 168.95, 187.35.

## 6. Refinement

Crystal data, data collection and structure refinement details are summarized in Table 3 ▸. The N-bound hydrogen atoms were located in a difference-Fourier map and refined freely [N10—H10 = 0.84 (3) and N13—H13 = 0.83 (3) Å]. The C-bound H atoms were positioned geometrically (C—H = 0.95 and 1.00 Å) and refined using a riding model with *U*_iso_(H) = 1.2 or 1.5*U*_eq_(C). The title compound was refined as an inversion twin with matrix [−1 0 0 0 − 1 0 0 0 − 1]; the resulting BASF value is 0.273 (14).

## Supplementary Material

Crystal structure: contains datablock(s) I. DOI: 10.1107/S205698902500670X/oi2024sup1.cif

Structure factors: contains datablock(s) I. DOI: 10.1107/S205698902500670X/oi2024Isup2.hkl

CCDC reference: 2476058

Additional supporting information:  crystallographic information; 3D view; checkCIF report

## Figures and Tables

**Table 1:** Hydrogen-bond geometry (Å, °) *Cg*1 is the centroid of the C4–C9 aromatic ring.

*D*—H⋯*A*	*D*—H	H⋯*A*	*D*⋯*A*	*D*—H⋯*A*
N10—H10⋯O3	0.84 (3)	2.34 (3)	2.804 (2)	115 (2)
N10—H10⋯O14^i^	0.84 (3)	2.30 (3)	3.105 (2)	161 (3)
N13—H13⋯O3	0.83 (3)	2.60 (3)	2.982 (2)	110 (2)
N13—H13⋯O11^i^	0.83 (3)	2.09 (3)	2.912 (2)	169 (3)
C1—H1⋯O11	1.00	2.26	2.746 (2)	108
C1—H1⋯O14	1.00	2.28	2.763 (2)	108
C5—H5⋯Cl1	0.95	2.77	3.179 (2)	107
C5—H5⋯Cl2	0.95	2.81	3.4098 (19)	122
C6—H6⋯O11^ii^	0.95	2.53	3.239 (3)	131
C12—H12*A*⋯Cl1^iii^	0.98	2.73	3.278 (3)	116
C12—H12*B*⋯O14^i^	0.98	2.51	3.407 (3)	152
C15—H15*B*⋯O11^i^	0.98	2.60	3.460 (3)	147
C15—H15*C*⋯*Cg*1^iv^	0.98	2.89	3.703 (3)	141

Symmetry codes: (i) 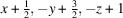; (ii) 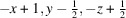; (iii) 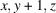; (iv) 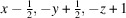.

**Table 2:** Summary of short inter­atomic contacts (Å)

Contact	Distance	Symmetry operation
Cl1⋯H12*A*	2.73	*x*, −1 + *y*, *z*
O14⋯H9	2.66	−1 + *x*, *y*, *z*
H6⋯O11	2.53	1 − *x*, − 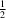 + *y*, 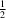 − *z*
H13⋯O11	2.09	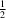 + *x*, 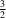 − *y*, 1 − *z*
C9⋯H6	3.08	2 − *x*, 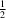 + *y*, 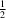 − *z*
C6⋯H15*B*	2.99	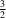 − *x*, 1 − *y*, − 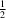 + *z*
H7⋯C4	3.08	2 − *x*, − 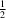 + *y*, 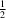 − *z*

**Table 3:** Experimental details

Crystal data	
Chemical formula	C_13_H_14_Cl_2_N_2_O_3_
*M*_r_	317.16
Crystal system, space group	Orthorhombic, *P*2_1_2_1_2_1_
Temperature (K)	100
*a*, *b*, *c* (Å)	8.9115 (5), 8.9341 (7), 18.5838 (15)
*V* (Å^3^)	1479.57 (19)
*Z*	4
Radiation type	Cu *K*α
μ (mm^−1^)	4.03
Crystal size (mm)	0.50 × 0.10 × 0.04

Data collection	
Diffractometer	XtaLAB Synergy, Dualflex, HyPix
Absorption correction	Gaussian (*CrysAlis PRO*; Rigaku OD, 2024 ▸)
*T*_min_, *T*_max_	0.225, 0.945
No. of measured, independent and observed [*I* > 2σ(*I*)] reflections	17780, 3244, 3199
*R*_int_	0.033
(sin θ/λ)_max_ (Å^−1^)	0.640

Refinement	
*R*[*F*^2^ > 2σ(*F*^2^)], *wR*(*F*^2^), *S*	0.025, 0.063, 1.12
No. of reflections	3244
No. of parameters	192
H-atom treatment	H atoms treated by a mixture of independent and constrained refinement
Δρ_max_, Δρ_min_ (e Å^−3^)	0.29, −0.35
Absolute structure	Refined as an inversion twin
Absolute structure parameter	0.273 (14)

Computer programs: *CrysAlis PRO* (Rigaku OD, 2024 ▸), *SHELXT2014/5* (Sheldrick, 2015*a* ▸), *SHELXL2018/3* (Sheldrick, 2015*b* ▸), *ORTEP-3 for Windows* (Farrugia, 2012 ▸) and *PLATON* (Spek, 2020 ▸).

## supplementary crystallographic information

### N-(2,2-Dichloro-1-acetamido-3-oxo-3-phenylpropyl)acetamide Crystal data

**Table:**

C_13_H_14_Cl_2_N_2_O_3_	*D*_x_ = 1.424 Mg m^−^^3^
*M**_r_* = 317.16	Cu *K*α radiation, λ = 1.54184 Å
Orthorhombic, *P*2_1_2_1_2_1_	Cell parameters from 13822 reflections
*a* = 8.9115 (5) Å	θ = 4.8–80.4°
*b* = 8.9341 (7) Å	µ = 4.03 mm^−^^1^
*c* = 18.5838 (15) Å	*T* = 100 K
*V* = 1479.57 (19) Å^3^	Needle, colourless
*Z* = 4	0.50 × 0.10 × 0.04 mm
*F*(000) = 656	

### N-(2,2-Dichloro-1-acetamido-3-oxo-3-phenylpropyl)acetamide Data collection

**Table:**

XtaLAB Synergy, Dualflex, HyPix diffractometer	3199 reflections with *I* > 2σ(*I*)
Radiation source: micro-focus sealed X-ray tube, PhotonJet (Cu) X-ray Source	*R*_int_ = 0.033
ω scans	θ_max_ = 80.7°, θ_min_ = 4.8°
Absorption correction: gaussian (CrysAlisPro; Rigaku OD, 2024)	*h* = −11→7
*T*_min_ = 0.225, *T*_max_ = 0.945	*k* = −11→11
17780 measured reflections	*l* = −23→23
3244 independent reflections	

### N-(2,2-Dichloro-1-acetamido-3-oxo-3-phenylpropyl)acetamide Refinement

**Table:**

Refinement on *F*^2^	Secondary atom site location: difference Fourier map
Least-squares matrix: full	Hydrogen site location: mixed
*R*[*F*^2^ > 2σ(*F*^2^)] = 0.025	H atoms treated by a mixture of independent and constrained refinement
*wR*(*F*^2^) = 0.063	*w* = 1/[σ^2^(*F*_o_^2^) + (0.025*P*)^2^ + 0.4827*P*] where *P* = (*F*_o_^2^ + 2*F*_c_^2^)/3
*S* = 1.12	(Δ/σ)_max_ = 0.001
3244 reflections	Δρ_max_ = 0.29 e Å^−^^3^
192 parameters	Δρ_min_ = −0.34 e Å^−^^3^
0 restraints	Absolute structure: Refined as an inversion twin
Primary atom site location: dual	Absolute structure parameter: 0.273 (14)

### N-(2,2-Dichloro-1-acetamido-3-oxo-3-phenylpropyl)acetamide Special details

**Table:**

Geometry. All esds (except the esd in the dihedral angle between two l.s. planes) are estimated using the full covariance matrix. The cell esds are taken into account individually in the estimation of esds in distances, angles and torsion angles; correlations between esds in cell parameters are only used when they are defined by crystal symmetry. An approximate (isotropic) treatment of cell esds is used for estimating esds involving l.s. planes.
Refinement. Refined as a two-component inversion twin

### N-(2,2-Dichloro-1-acetamido-3-oxo-3-phenylpropyl)acetamide Fractional atomic coordinates and isotropic or equivalent isotropic displacement parameters (Å^2^)

**Table:**

	*x*	*y*	*z*	*U*_iso_*/*U*_eq_	
C1	0.4571 (2)	0.6512 (2)	0.42758 (11)	0.0153 (4)	
H1	0.350530	0.632488	0.413306	0.018*	
C2	0.5571 (2)	0.5762 (2)	0.36927 (11)	0.0164 (4)	
C3	0.7281 (2)	0.5941 (2)	0.38261 (11)	0.0150 (4)	
C4	0.8415 (2)	0.5028 (2)	0.34286 (11)	0.0166 (4)	
C5	0.8137 (2)	0.4219 (2)	0.27994 (12)	0.0193 (4)	
H5	0.717015	0.424755	0.258427	0.023*	
C6	0.9271 (3)	0.3374 (3)	0.24883 (12)	0.0223 (4)	
H6	0.908252	0.283161	0.205781	0.027*	
C7	1.0687 (2)	0.3319 (3)	0.28069 (13)	0.0241 (4)	
H7	1.145748	0.272622	0.259784	0.029*	
C8	1.0970 (2)	0.4128 (3)	0.34284 (14)	0.0245 (5)	
H8	1.193639	0.408985	0.364383	0.029*	
C9	0.9849 (2)	0.4996 (2)	0.37380 (11)	0.0197 (4)	
H9	1.005393	0.556575	0.415835	0.024*	
C11	0.3557 (2)	0.9022 (2)	0.42317 (11)	0.0180 (4)	
C12	0.3834 (3)	1.0663 (3)	0.43368 (15)	0.0276 (5)	
H12A	0.359605	1.120063	0.389145	0.041*	
H12B	0.489068	1.082549	0.446033	0.041*	
H12C	0.319644	1.103611	0.472727	0.041*	
C14	0.3606 (2)	0.5148 (2)	0.53082 (12)	0.0183 (4)	
C15	0.3939 (3)	0.4528 (3)	0.60425 (13)	0.0263 (5)	
H15A	0.321531	0.492994	0.638963	0.039*	
H15B	0.495648	0.481658	0.618601	0.039*	
H15C	0.386103	0.343439	0.603080	0.039*	
Cl1	0.51039 (5)	0.38234 (5)	0.36798 (3)	0.02095 (12)	
Cl2	0.51459 (6)	0.65975 (6)	0.28413 (3)	0.02384 (12)	
N10	0.47663 (19)	0.81155 (19)	0.42972 (9)	0.0165 (3)	
N13	0.47820 (19)	0.58088 (19)	0.49669 (9)	0.0155 (3)	
O3	0.76361 (16)	0.68281 (18)	0.42875 (9)	0.0206 (3)	
O11	0.22974 (16)	0.85474 (18)	0.40820 (9)	0.0205 (3)	
O14	0.23568 (17)	0.50255 (19)	0.50325 (8)	0.0213 (3)	
H10	0.559 (3)	0.845 (3)	0.4435 (15)	0.014 (6)*	
H13	0.557 (3)	0.600 (3)	0.5189 (15)	0.016 (6)*	

### N-(2,2-Dichloro-1-acetamido-3-oxo-3-phenylpropyl)acetamide Atomic displacement parameters (Å^2^)

**Table:**

	*U*^11^	*U*^22^	*U*^33^	*U*^12^	*U*^13^	*U*^23^
C1	0.0103 (8)	0.0181 (9)	0.0175 (9)	0.0000 (7)	0.0001 (7)	0.0008 (7)
C2	0.0128 (8)	0.0198 (9)	0.0167 (9)	−0.0005 (7)	−0.0013 (7)	0.0016 (8)
C3	0.0133 (8)	0.0167 (8)	0.0150 (9)	−0.0015 (7)	−0.0004 (7)	0.0013 (7)
C4	0.0147 (9)	0.0173 (9)	0.0178 (9)	−0.0003 (7)	0.0018 (7)	0.0013 (8)
C5	0.0175 (9)	0.0226 (10)	0.0179 (10)	−0.0011 (8)	−0.0010 (8)	−0.0005 (8)
C6	0.0236 (10)	0.0247 (10)	0.0186 (10)	0.0007 (8)	0.0029 (8)	−0.0031 (9)
C7	0.0191 (9)	0.0271 (11)	0.0260 (11)	0.0035 (8)	0.0055 (8)	−0.0030 (10)
C8	0.0133 (9)	0.0304 (11)	0.0298 (12)	0.0023 (8)	−0.0006 (8)	−0.0017 (10)
C9	0.0138 (9)	0.0232 (9)	0.0221 (9)	−0.0019 (8)	0.0005 (8)	−0.0023 (8)
C11	0.0160 (9)	0.0213 (10)	0.0166 (9)	0.0022 (8)	0.0017 (7)	0.0028 (8)
C12	0.0234 (10)	0.0201 (10)	0.0393 (13)	0.0033 (8)	−0.0041 (10)	0.0040 (9)
C14	0.0149 (9)	0.0187 (9)	0.0212 (10)	−0.0006 (7)	0.0013 (8)	−0.0029 (8)
C15	0.0228 (10)	0.0342 (12)	0.0219 (11)	−0.0067 (9)	−0.0016 (9)	0.0056 (9)
Cl1	0.0161 (2)	0.0188 (2)	0.0279 (2)	−0.00432 (17)	0.00332 (18)	−0.00562 (17)
Cl2	0.0204 (2)	0.0342 (3)	0.0169 (2)	0.0074 (2)	−0.00126 (18)	0.00409 (19)
N10	0.0106 (7)	0.0173 (7)	0.0215 (8)	−0.0015 (6)	0.0007 (6)	0.0006 (6)
N13	0.0102 (7)	0.0186 (8)	0.0177 (8)	−0.0009 (6)	−0.0009 (7)	−0.0004 (6)
O3	0.0133 (6)	0.0228 (7)	0.0255 (8)	−0.0005 (6)	−0.0006 (6)	−0.0061 (6)
O11	0.0126 (6)	0.0258 (7)	0.0231 (7)	0.0017 (6)	−0.0009 (5)	0.0006 (6)
O14	0.0129 (7)	0.0267 (7)	0.0244 (8)	−0.0045 (6)	−0.0007 (6)	0.0006 (6)

### N-(2,2-Dichloro-1-acetamido-3-oxo-3-phenylpropyl)acetamide Geometric parameters (Å, º)

**Table:**

C1—N13	1.442 (3)	C8—C9	1.389 (3)
C1—N10	1.444 (3)	C8—H8	0.9500
C1—C2	1.555 (3)	C9—H9	0.9500
C1—H1	1.0000	C11—O11	1.232 (3)
C2—C3	1.552 (3)	C11—N10	1.354 (3)
C2—Cl1	1.781 (2)	C11—C12	1.499 (3)
C2—Cl2	1.790 (2)	C12—H12A	0.9800
C3—O3	1.210 (3)	C12—H12B	0.9800
C3—C4	1.494 (3)	C12—H12C	0.9800
C4—C5	1.397 (3)	C14—O14	1.230 (3)
C4—C9	1.402 (3)	C14—N13	1.360 (3)
C5—C6	1.388 (3)	C14—C15	1.502 (3)
C5—H5	0.9500	C15—H15A	0.9800
C6—C7	1.395 (3)	C15—H15B	0.9800
C6—H6	0.9500	C15—H15C	0.9800
C7—C8	1.386 (4)	N10—H10	0.84 (3)
C7—H7	0.9500	N13—H13	0.83 (3)
			
N13—C1—N10	113.07 (17)	C9—C8—H8	119.8
N13—C1—C2	111.00 (16)	C8—C9—C4	119.8 (2)
N10—C1—C2	112.19 (16)	C8—C9—H9	120.1
N13—C1—H1	106.7	C4—C9—H9	120.1
N10—C1—H1	106.7	O11—C11—N10	122.7 (2)
C2—C1—H1	106.7	O11—C11—C12	121.08 (19)
C3—C2—C1	114.02 (17)	N10—C11—C12	116.25 (19)
C3—C2—Cl1	109.38 (14)	C11—C12—H12A	109.5
C1—C2—Cl1	107.12 (13)	C11—C12—H12B	109.5
C3—C2—Cl2	107.82 (14)	H12A—C12—H12B	109.5
C1—C2—Cl2	108.36 (13)	C11—C12—H12C	109.5
Cl1—C2—Cl2	110.13 (11)	H12A—C12—H12C	109.5
O3—C3—C4	122.06 (18)	H12B—C12—H12C	109.5
O3—C3—C2	115.96 (18)	O14—C14—N13	122.8 (2)
C4—C3—C2	121.94 (18)	O14—C14—C15	121.6 (2)
C5—C4—C9	119.65 (19)	N13—C14—C15	115.55 (19)
C5—C4—C3	125.17 (18)	C14—C15—H15A	109.5
C9—C4—C3	115.17 (18)	C14—C15—H15B	109.5
C6—C5—C4	120.07 (19)	H15A—C15—H15B	109.5
C6—C5—H5	120.0	C14—C15—H15C	109.5
C4—C5—H5	120.0	H15A—C15—H15C	109.5
C5—C6—C7	120.1 (2)	H15B—C15—H15C	109.5
C5—C6—H6	120.0	C11—N10—C1	119.69 (17)
C7—C6—H6	120.0	C11—N10—H10	120.8 (19)
C8—C7—C6	120.0 (2)	C1—N10—H10	118.2 (19)
C8—C7—H7	120.0	C14—N13—C1	120.26 (17)
C6—C7—H7	120.0	C14—N13—H13	120.6 (19)
C7—C8—C9	120.4 (2)	C1—N13—H13	117 (2)
C7—C8—H8	119.8		
			
N13—C1—C2—C3	−64.2 (2)	C9—C4—C5—C6	0.8 (3)
N10—C1—C2—C3	63.4 (2)	C3—C4—C5—C6	−178.1 (2)
N13—C1—C2—Cl1	56.97 (18)	C4—C5—C6—C7	0.6 (3)
N10—C1—C2—Cl1	−175.45 (13)	C5—C6—C7—C8	−1.1 (4)
N13—C1—C2—Cl2	175.76 (13)	C6—C7—C8—C9	0.1 (4)
N10—C1—C2—Cl2	−56.66 (18)	C7—C8—C9—C4	1.3 (4)
C1—C2—C3—O3	−11.6 (3)	C5—C4—C9—C8	−1.7 (3)
Cl1—C2—C3—O3	−131.54 (17)	C3—C4—C9—C8	177.2 (2)
Cl2—C2—C3—O3	108.71 (19)	O11—C11—N10—C1	−7.4 (3)
C1—C2—C3—C4	166.04 (18)	C12—C11—N10—C1	173.8 (2)
Cl1—C2—C3—C4	46.2 (2)	N13—C1—N10—C11	−108.3 (2)
Cl2—C2—C3—C4	−73.6 (2)	C2—C1—N10—C11	125.26 (18)
O3—C3—C4—C5	−166.3 (2)	O14—C14—N13—C1	5.0 (3)
C2—C3—C4—C5	16.2 (3)	C15—C14—N13—C1	−176.70 (19)
O3—C3—C4—C9	14.8 (3)	N10—C1—N13—C14	113.2 (2)
C2—C3—C4—C9	−162.76 (19)	C2—C1—N13—C14	−119.66 (19)

### N-(2,2-Dichloro-1-acetamido-3-oxo-3-phenylpropyl)acetamide Hydrogen-bond geometry (Å, º)

**Table:**

*D*—H···*A*	*D*—H	H···*A*	*D*···*A*	*D*—H···*A*
N10—H10···O3	0.84 (3)	2.34 (3)	2.804 (2)	115 (2)
N10—H10···O14^i^	0.84 (3)	2.30 (3)	3.105 (2)	161 (3)
N13—H13···O3	0.83 (3)	2.60 (3)	2.982 (2)	110 (2)
N13—H13···O11^i^	0.83 (3)	2.09 (3)	2.912 (2)	169 (3)
C1—H1···O11	1.00	2.26	2.746 (2)	108
C1—H1···O14	1.00	2.28	2.763 (2)	108
C5—H5···Cl1	0.95	2.77	3.179 (2)	107
C5—H5···Cl2	0.95	2.81	3.4098 (19)	122
C6—H6···O11^ii^	0.95	2.53	3.239 (3)	131
C12—H12*A*···Cl1^iii^	0.98	2.73	3.278 (3)	116
C12—H12*B*···O14^i^	0.98	2.51	3.407 (3)	152
C15—H15*B*···O11^i^	0.98	2.60	3.460 (3)	147
C15—H15*C*···*Cg*1^iv^	0.98	2.89	3.703 (3)	141

Symmetry codes: (i) *x*+1/2, −*y*+3/2, −*z*+1; (ii) −*x*+1, *y*−1/2, −*z*+1/2; (iii) *x*, *y*+1, *z*; (iv) *x*−1/2, −*y*+1/2, −*z*+1.
